# Cardiomyocyte-targeting exosomes from sulforaphane-treated fibroblasts affords cardioprotection in infarcted rats

**DOI:** 10.1186/s12967-023-04155-x

**Published:** 2023-05-09

**Authors:** Gaia Papini, Giulia Furini, Marco Matteucci, Vanessa Biemmi, Valentina Casieri, Nicole Di Lascio, Giuseppina Milano, Lucia Rosa Chincoli, Francesco Faita, Lucio Barile, Vincenzo Lionetti

**Affiliations:** 1 Unit of Translational Critical Care Medicine, Laboratory of Basic and Applied Medical Sciences, The Interdisciplinary Research Center “Health Science”, Scuola Superiore Sant’Anna, Via G. Moruzzi, 1, 56124 Pisa, Italy; 2 Cardiovascular Theranostics, Istituto Cardiocentro Ticino, Laboratories for Translational Research, Ente Ospedaliero Cantonale, Bellinzona, Switzerland; 3 Department of Life Sciences, University of Siena, Siena, Italy; 4 Institute of Clinical Physiology, CNR, Pisa, Italy; 5 Faculty of Biomedical Sciences, Università Svizzera Italiana, 6900 Lugano, Switzerland; 6 Anesthesiology and Intensive Care Medicine, UOSVD, Fondazione Toscana G. Monasterio, Pisa, Italy

**Keywords:** Cardioprotection, Exosomes, Allogeneic fibroblast, Remodeling, Myocardial infarction, Sulforaphane (PubChem CID: 5350)

## Abstract

**Background:**

Exosomes (EXOs), tiny extracellular vesicles that facilitate cell–cell communication, are being explored as a heart failure treatment, although the features of the cell source restrict their efficacy. Fibroblasts the most prevalent non-myocyte heart cells, release poor cardioprotective EXOs. A noninvasive method for manufacturing fibroblast-derived exosomes (F-EXOs) that target cardiomyocytes and slow cardiac remodeling is expected. As a cardioprotective isothiocyanate, sulforaphane (SFN)-induced F-EXOs (SFN-F-EXOs) should recapitulate its anti-remodeling properties.

**Methods:**

Exosomes from low-dose SFN (3 μM/7 days)-treated NIH/3T3 murine cells were examined for number, size, and protein composition. Fluorescence microscopy, RT-qPCR, and western blot assessed cell size, oxidative stress, AcH4 levels, hypertrophic gene expression, and caspase-3 activation in angiotensin II (AngII)-stressed HL-1 murine cardiomyocytes 12 h-treated with various EXOs. The uptake of fluorescently-labeled EXOs was also measured in cardiomyocytes. The cardiac function of infarcted male Wistar rats intramyocardially injected with different EXOs (1·10^12^) was examined by echocardiography. Left ventricular infarct size, hypertrophy, and capillary density were measured.

**Results:**

Sustained treatment of NIH/3T3 with non-toxic SFN concentration significantly enhances the release of CD81 + EXOs rich in TSG101 (Tumor susceptibility gene 101) and Hsp70 (Heat Shock Protein 70), and containing maspin, an endogenous histone deacetylase 1 inhibitor. SFN-F-EXOs counteract angiotensin II (AngII)-induced hypertrophy and apoptosis in murine HL-1 cardiomyocytes enhancing SERCA2a (sarcoplasmic/endoplasmic reticulum Ca^2+^ ATPase 2a) levels more effectively than F-EXOs. In stressed cardiomyocytes, SFN-F-EXOs boost AcH4 levels by 30% (p < 0.05) and significantly reduce oxidative stress more than F-EXOs. Fluorescence microscopy showed that mouse cardiomyocytes take in SFN-F-EXOs ~ threefold more than F-EXOs. Compared to vehicle-injected infarcted hearts, SFN-F-EXOs reduce hypertrophy, scar size, and improve contractility.

**Conclusions:**

Long-term low-dose SFN treatment of fibroblasts enhances the release of anti-remodeling cardiomyocyte-targeted F-EXOs, which effectively prevent the onset of HF. The proposed method opens a new avenue for large-scale production of cardioprotective exosomes for clinical application using allogeneic fibroblasts.

**Supplementary Information:**

The online version contains supplementary material available at 10.1186/s12967-023-04155-x.

## Introduction

Myocardial infarction (MI) remains the leading cause of heart failure (HF) and death worldwide [1]. Currently, there is a lot of interest in developing innovative approaches to early prevent post-MI left ventricular (LV) remodeling. Indeed, because it involves several cardiac cell types simultaneously exposed to an ischemic milieu, adverse LV remodeling is difficult to effectively counteract [2]. At the moment, fibroblast (F)-mediated intercellular communication is a viable therapeutic target for preventing the onset of HF following an insult [3].

The angiotensin II (AngII) signaling pathway affects fibroblast-cardiomyocyte cross-talk [4] and its post-ischemic activation [5] leads to oxidative stress [6], apoptosis [7] and hypertrophic growth in surviving cardiomyocytes [8, 9]. Otherwise, AngII-mediated fibroblasts activation in infarcted heart results in focal replacement fibrosis [9]. Although numerous drugs have been identified to delay the onset and progression of HF, potential systemic side effects and a lack of clinical efficacy in nonresponsive individuals throw their usage into question [10]. As a result, the development of precision anti-remodeling cardiac therapy should focus on optimizing fibroblast-cardiomyocyte interplay in each clinical setting, including during the perioperative phase [11].

Exosomes (EXOs) are important vesicular mediators of intercellular communication in cardiac muscle [12] and their cargo has demonstrated an high potential for activating many endogenous cardioprotective mechanisms, even in surgical [11] and critically ill patients [13]. Exosomes are released by all living cardiac cells in response to varied stimuli [12], but their intrinsic tropism for specific target cells [14] can influence EXOs uptake efficiency and biological effects in varying magnitude ranges under distinct pathophysiological scenarios. It is well-known that fibroblasts are a good source of exosomes (F-EXOs) which make cardiomyocytes susceptible to AngII-induced maladaptive hypertrophy [15, 16]. Furthermore, F-EXOs isolated from HF patients elicit a pathogenic phenotype in cultured cardiomyocytes [17]. In this regard, we found that EXOs released by healthy cardiac fibroblasts have neither effective anti-apoptotic and angiogenic effects in infarcted hearts [18, 19]. However, one study comes to the opposite result [20]. Although cardiac transplantation of exosomes is an efficient cell-free therapeutic strategy for heart repair [12], the above-mentioned controversial results discourage the use of fibroblasts as a source of exosomes for life-saving treatment. However, because fibroblasts are easily isolated and cultured versatile cells, finding a way to produce adaptive exosomes from them is clinically relevant.

Previous studies have shown that exogenous bioactive compounds, regardless of parent cells, regulate biogenesis, cargo, release and uptake of cardioprotective exosomes [21, 22]. Therefore, the identification of safe and effective conditioning agent is critical for the release of a substantial amount of cardioprotective exosomes with high tropism toward cardiomyocytes from allogeneic fibroblasts in an ischemic microenvironment.

Edible plant-derived chemicals are biologically active and provide cardioprotection by preventing, slowing or reversing cardiac remodeling [23]. At lower doses, sulforaphane (SFN), the most thoroughly researched hormetic dietary isothiocyanate, reversed remodeling and enhanced contractility of infarcted hearts [24]. As a result, we investigated whether low-dose SFN stimulates the release of cardiomyocyte-targeted exosomes from healthy fibroblasts. Primary cardiac fibroblasts are isolated from cardiac biopsy and are difficult to keep alive in cell culture for lengthy periods of time, impeding physiologically relevant investigations [25]. In our proof-of-principle study, we tested SFN in NIH/3T3 mouse embryonic fibroblasts, which mimic the response of adult cardiac fibroblasts [26]. It is a reliable in vitro model for evaluating the effects of pharmaceutical substances and phytochemicals, to the best of our knowledge. Cultured NIH/3T3 fibroblasts have been utilized successfully to assess SFN’s dose-dependent antioxidant effects [27]. Exosomes released from SFN-treated fibroblasts (SFN-F-EXOs) were first tested for anti-hypertrophic, anti-apoptotic and pro-angiogenic in murine adult HL-1 cardiomyocytes [28, 29] and cardiac endothelial cells (MCEC) [30] chronically exposed to AngII. We also looked at exosome uptake by recipient cells. Finally, we tested SFN-F-EXOs for in vivo cardioprotection in an established rat model of non-reperfused MI [18, 19, 31, 32].

## Methods

### Chemical reagents

AngII (Cat. A9525), dimethyl sulfoxide (DMSO) (Cat. D8418) and L-SFN (PubChem CID: 5350) (Cat. S6317) were purchased from Sigma-Aldrich Chemical Co, Merk Life Science S.r.l., Milan, Italy. In accordance with the manufacturer’s instructions, SFN stock solution (48 mM) was prepared in DMSO, aliquoted and stored at − 20 °C. AngII stock solution (1 mM) was prepared in sterile distilled water, aliquoted and kept at − 20 °C. Repeated freeze–thaw cycles were avoided, and stock solutions were only stored for 6 weeks. The stock solutions were further diluted with the appropriate complete culture medium.

### Cell lines

Mouse NIH/3T3 embryo fibroblasts (ATCC® CRL-1658™ kindly provided by Dr. Giuseppe Rainaldi, IFC-CNR, Pisa, Italy) are frequently used as in vitro models of adult cardiac fibroblasts [33, 34] and fibroblast-cardiomyocytes exosome-based crosstalk [35]. HL-1 mouse atrial cardiomyocytes (a kind gift of Professor W. Claycomb, LSUHSC, New Orleans, LA, USA), a cell line used to study cardiac physiology at the cellular level [36] and to assess the cardioprotective effects of exosomes [18, 19, 21, 37, 38], were cultured in Claycomb Medium (Sigma-Aldrich, Merk Life Science S.r.l., Milan, Italy) [39] supplemented with 10% exosomes-depleted FBS, 100units/mL of penicillin, 100 µg/mL streptomycin, 250 ng/mL amphotericin B and 2 mM L-glutamine (Sigma-Aldrich, Merk Life Science S.r.l., Milan, Italy). Murine immortalized cardiac endothelial cells (MCEC; Tebu-bio Srl, Magenta, Italy) were maintained in Dulbecco’s Modified Eagle’s Medium (DMEM, Sigma-Aldrich, Merk Life Science S.r.l., Milan, Italy) containing 1 g/L glucose and supplemented with 10% (V/V) fetal bovine serum (FBS) (System Biosciences, Palo Alto, CA, USA), 100units/mL of penicillin, 100 µg/mL streptomycin, 250 ng/mL amphotericin B and 2 mM L-glutamine.

### Cell treatments

For 7 days (d), fibroblasts were treated with increasing doses of SFN (1.5—48uM) or 0.01% DMSO in complete medium in order to produce SFN-F-EXOs. To mimic an ischemic microenvironment, HL-1 [40] and MCEC [41] cells were treated with 100 nM AngII (Sigma-Aldrich, Merk Life Science S.r.l., Milan, Italy) for at least 24 h. The treatment was carried out in exosome-free media, which consists of the appropriate growth medium supplemented with 10%(v/v) exosome-depleted FBS (System Biosciences, Palo Alto, CA, USA) whenever exosomes were isolated from cell-conditioned medium.

### Cell viability assay

According to the manufacturer, the MTT test was used to assess the relative number of viable cells after each treatment. In brief, 1,000 fibroblasts/well were plated on a 96-well plate and allowed to adhere overnight before being treated with increasing dosages of SFN or DMSO (3 or 7d) the next day. After treatment, the culture media was removed, cells were washed with phosphate buffered saline (PBS) before being treated for 4 h at 37 °C with 500 μg/mL 3-(4,5-Dimethylthiazol-2-yl)-2,5-Diphenyltetrazolium Bromide (MTT, Sigma-Aldrich, Merk Life Science S.r.l., Milan, Italy). MTT was removed, and purple formazan crystals were dissolved in acidified isopropanol (100% isopropanol containing 0.31% (v/v) HCl) under steady agitation for 10 min (min) at room temperature (RT). A conventional plate-reader spectrophotometer was used to measure absorbance at 540 nm (signal) and 660 nm (background).

### Exosomes isolation and quantification

Exosomes were isolated from normal and SFN-treated NIH/3T3 conditioned medium using a differential centrifugation protocol previously described by us [18, 19, 21] and in compliance with the most recent Minimal Information for Studies of Extracellular Vesicles (“MISEV”) guideline [42].

As previously described by us [18, 19, 21, 37, 38], exosome pools were investigated using a NanoSight LM10 nanoparticle tracking analysis (NTA) device (NanoSight Ltd, UK) to evaluate particle size and concentration. In a nutshell, the exosome pellet was re-suspended in 100μL of filtered (0.2 μm filter) PBS. Prior to analysis, the exosome suspension was diluted ten times in PBS to match the instrument’s optimal detection range. The daily rate of exosome release was calculated by dividing the number of exosomes isolated from a P-100 plate (quantified by NTA) by the 70,000 initial cells seeded and the 7d-treatment duration.

### In vitro experimental protocols

HL-1 and MCEC were planted in either 8-well chamber slides (for fluorescence-based experiments) or 6-well plates (for protein or RNA extraction) depending on the assay. After 12 h, the culture medium was replaced with exosome-free complete growth medium with or without 100 nM AngII, and the cells were incubated for another 12 h. In accordance with the experimental protocol of each assay, a single dose of F-EXOs or SFN-F-EXOs suspended in similar volume of sterile PBS (100µL) was added directly to each well, and cells were incubated for additional time depending on specific assay, maintaining AngII in the culture medium of stressed cells. As a control, just PBS was used.

### Cell size measurement by cytoskeletal staining

HL-1 cardiomyocytes were seeded in an 8-well chamber slide (8000 cells/well), allowed to adhere for 12 h, and then treated with/without 100 nM AngII for 12 h as previously described. Each well received 30 µg of F-EXOs or SFN-F-EXOs, and cells were cultured for another 12 h. As a non-treated control, PBS was added alone. The cell monolayer was rinsed with PBS the next day, fixed (4% paraformaldehyde, PFA, 15 min at room temperature) and permeabilized (0.1% TritonX-100, 2 × 2 min). To stain the filamentous actin of the cytoskeleton, cells were incubated for 1 h at RT with 1:300 dilution of Phalloidin-Atto 550 (Sigma-Aldrich, Merk Life Science S.r.l., Milan, Italy). PBS was used to remove excess dye before mounting the gass slide with DAPI-containing Vectashield (DBA Italia srl, Segrate, Italy). The images were acquired using a fluorescence microscope (DM2500; Leica Microsystems srl, Milan, Italy) (20 × magnification). ImageJ software was used to manually quantify cell area, which was then expressed as pixels.

### ROS detection

Superoxide (O2-) generation was measured by staining with 5 mol/L dihydroethidium (DHE, Invitrogen™, Thermo Fisher Scientific, Waltham, MA, USA) in PBS for 30 min at 37 °C [43]. HL-1 cardiomyocytes were seeded in an 8-well chamber slide (8,000 cells/well), allowed to adhere for 12 h, and then treated with/without 100 nM AngII for 12 h as previously described. Each well received 30 μg of F-EXOs or SFN-F-EXOs, and cells were cultured for another 12 h with/without 100 nM AngII. PBS alone was added as a control. The cell monolayer was washed three times with PBS before being mounted on glass slides were with DAPI-containing Vectashield (DBA Italia srl, Segrate, Italy). A fluorescence microscope (DM2500; Leica Microsystems srl, Milan, Italy) was used to capture the images (20 ×magnification). ImageJ software was used to quantify the fluorescence intensity of DHE.

### Immunofluorescent staining of Acetylated H4 histone

HL-1 cardiomyocytes were seeded in an 8-well chamber slide (8000 cells/well), allowed to adhere for 12 h, and then treated with/without 100 nM AngII for 12 h as previously described. Each well received 30 µg of F-EXOs or SFN-F-EXOs, and cells were cultured for another 12 h. As a control, PBS was added alone. Acetylated histone H4 (AcH4) was stained with an antibody against acetylated H4 histone (Millipore) (overnight, at 4 °C), followed by Alexa-Fluor488 secondary antibody (Abcam, Cambridge, UK; 1:300). Images were acquired using a fluorescence microscope (DM2500; Leica Microsystems srl) (20 × magnification). Cells were fixed with 4% (w/v) PFA in PBS (10 min at RT) and permeabilized with 0.1% Triton-X100 (5 min at RT), with PBS washed in between steps. The cells were blocked in 1% (w/v) Bovine Serum Albumin (Sigma-Aldrich, Merk Life Science S.r.l., Milan, Italy) (1 h at RT). Fluorescence intensity was quantified with ImageJ software.

### Cellular uptake of fluorescently-labelled exosomes

To investigate exosome uptake from cardiac cells, HL-1 and MCEC cells were seeded in an 8-well chamber slide (15,000 cells/well), adhered for 12 h, and then pre-treated with/without 100 nM AngII for 12 h as previously described. The exosomal pellet obtained by ultracentrifugation was first fluorescently labeled by overnight incubation at 4 °C in the dark with constant shaking with a 1:1000 dilution of the lipophilic probe 4-(4-(Dihexadecylamino)styryl)-N-Methylpyridinium Iodide (DiA, Invitrogen™, Thermo Fisher Scientific, Waltham, MA, USA), in accordance with the manufacturer’s guidelines. Ultracentrifugation was used to remove the excess dye. After that, 100 µg of labeled exosomes were added to each chamber well and cells were cultured for another 3 h. After incubation, the media was removed, and the cells were washed with PBS and fixed for 10 min at RT with 4% (w/v) PFA. The cell monolayer was washed three times with PBS before being mounted on glass slides with DAPI containing Vectashield (DBA Italia srl, Segrate, Italy). Cells were observed with a fluorescence microscope (DM2500; Leica Microsystems srl, Milan, Italy) at 20 × magnification. Exosomal uptake was quantified as the ratio between DiA fluorescence to cell surface area using ImageJ software.

### Western blot

To extract cellular proteins, cells were detached with trypsin, pelleted by centrifugation (900 rpm for 5 min), washed once in PBS and re-suspended in 100μL radiommunoprecipitation assay (RIPA) buffer [50 mM Tris, 300 mM NaCl, 5mMEDTA, 1% (v / v) NP40, 0,1% (w/v) SDS, 0,5% (p/v) of sodium deoxycholate] containing protease inhibitors (Sigma-Aldrich, Merk Life Science S.r.l., Milan, Italy). Cells lysates were incubated on ice for 30 min before being centrifuged at 10,000 g at 4 °C for 10 min to precipitate undigested cell membranes and collecting the supernatant (clear total cell lysate). To extract exosomal proteins, the ultracentrifuged F-EXO pellet was resuspended in 50μL RIPA buffer with protease inhibitors. After 30 min, the samples were sonicated three times on ice for 5 min each to increase digestion efficiency. The BCA protein assay (Thermo Fisher Scientific, Waltham, MA, USA) was used to determine protein concentration. SDS-PAGE was used to separate equal amounts of proteins on 10–15% (w/v) acrylamide-bis acrylamide gels under reducing and denaturing conditions. Proteins were transferred to a nitrocellulose membrane (Bio-Rad Laboratories, Inc., Italy) and probed overnight at 4 °C with the appropriate primary antibodies. Primary antibodies towards caspase 3 (Santa Cruz Biotechnology Inc., USA, sc-7148, lot number: J2815, 1:500), acetylated H4 histone (Millipore, Merk Life Science S.r.l., Milan, Italy, 06–866, lot number: 2664262, 1:1000), total H4 histone (Abcam, Cambridge, UK, ab10158, lot number: GR194493-1 and GR114730-1, 1:500), acetylated H3 histone (Acetyl K14) (Abcam, Cambridge, UK, Ab52946, lot number: GR149741-20, 1:1000), total H3 histone (Cell Signaling Technology Inc., Euroclone S.p.A., Pero, Italy, 4499, lot number: Lot-1 and Lot-9, 1:500), maspin (Santa Cruz Biotechnology Inc., USA sc-8543, lot number: D1009, 1:200), nuclear factor erythroid 2–related factor 2 (Nrf2) (Abcam, Cambridge, UK, Ab31163, lot number: GR298097-3, 1:500), heat shock protein 70 (Hsp70) (Enzo Life Sciences, Euroclone S.p.A., Pero, Italy, ADI-SPA-812F, lot number: 8101522, 1:500; Abcam, Cambridge, UK, Ab2787, GR3253215-4, 1:1000), CD81 (Santa Cruz Biotechnology, Inc., USA, sc-166029, lot number: F2416, 1:100), tumor susceptibility gene 101 (TSG101) (Sigma-Aldrich, Merk Life Science S.r.l., Milan, Italy, T5701, lot number: 037M4775V, 1:1000) and β-tubulin (Sigma-Aldrich, Merk Life Science S.r.l., Milan, Italy, T5201, lot number: 015M4819V, 1:500) were employed in this study. After incubation with the appropriate horseradish peroxidase-conjugated secondary antibodies (Sigma-Aldrich, Merk Life Science S.r.l., Milan, Italy) for 1 h at RT, immunoreactive bands were detected by chemiluminescence (ECL substrate, Thermo Fisher Scientific, Waltham, MA, USA). In parallel, we used reversible Ponceau staining to ensure that the gels were loaded evenly.

### Real-time quantitative reverse transcription PCR

RNA was extracted from cultured cells in order to evaluate the expression of certain genes using quantitative real-time PCR (RT-qPCR). Following the manufacturer's instructions, RNA was extracted using the AurumTM Total RNA Mini Kit (Bio-Rad Laboratories, Inc., Italy); 1 µg of RNA was retrotranscribed using the PrimeScript™ RT reagent kit with genomic DNA removal (Takara Bio, Saint-Germain-en-Laye, France). The cDNA was diluted 10 times in nuclease-free water before being used in qPCR. The qPCR was performed on 5µL-diluted cDNA using the QuantiTect SYBR Green PCR Master Mix (Qiagen, Milan, Italy) and according to the manufacturers’ instructions. The primers listed below were used: brain natriuretic peptide (BNP) FW = 5′-CGTTTGGGCTGTAACGCACT-3′, RV = 5′-TCACTTCAAAGGTGGTCCCAG-3′; sarcoplasmic/endoplasmic reticulum calcium (Ca2 +)-ATPase cardiac isoform 2a (SERCA2a) FW = 5′-CCTTTGCCGCTCATTTTCCAG-3′, RV = 5′-GGCTGCACACACTCTTTACC-3′; β-Actin FW = 5′-GGCACCACACCTTCTACAATG-3′, RV = 5′-GGGGTGTTGAAGGTCTCAAAC-3′. All primers were purchased from Sigma-Aldrich and used at a concentration of 500 nm. In accord with MIQE guidelines, relative quantification of transcript expression was performed using the 2-ΔΔCt comparative method [44]. β-Actin was used as housekeeping gene.

### Rat model of myocardial infarction

Twenty five healthy male RccHan^®^ Wistar outbred rats (10–12 weeks old, 200–300 g body weight) were subjected to non-reperfused myocardial infarction (MI) by closure of the left anterior descending coronary artery (LAD), as previously described by us [18, 19, 31, 32]. In brief, the overnight fasted animals were anesthetized with an intraperitoneal injection of ketamine (300 μl/animal, Ketanarkon^®^ 100; Streuli AG) + xylazine (15 μl/animal, Streuli AG), intubated, and mechanically ventilated while ECG and body temperature were monitored. 1–2% isoflurane (in 60% air, 40% oxygen) was used to maintain general anesthesia. The LAD was permanently tied off near its emergence (~ 2 mm) below the left atrium with 6–0 silk. Five rats (20%) died immediately after coronary ligation as a result of persistent ventricular fibrillation. Following coronary ligation, the surviving animals' left ventricular region bordering the infarcted area was injected three times with 100μL PBS (n = 7, vehicle), F-EXOs (n = 7, 10^12^ nanoparticles), or SFN-F-EXOs (n = 6, 10^12^ nanoparticles) before chest closure and suture. The quantity of exosomes to inject was determined by the number of nanovesicles measured per fibroblast in our tests, as well as the relative number of fibroblasts compared to other cardiac cell types [45], and it is consistent with earlier research that used exosome injection in rat hearts [19].

The surgical pain was controlled by daily administration of Meloxicam (2 mg/kg subcutaneus initial dose followed by 1 mg/kg subcutaneous for 2d, Mobic, Boehringer Ingelheim Pharma GmbH & Co KG, Biberach Germany). Rats were anesthetized with 2% isoflurane and sacrificed by lethal potassium chloride injection, which caused diastolic arrest of cardiac activity, 28 days after MI.

### Transthoracic echocardiography

Transthoracic echocardiography was performed on fasting infarcted rats that had been sedated (14 mg/kg Xylazine + 40 mg/kg Zoletil 100 /animal; intraperitoneal) (PBS, n = 5; F-EXOs, n = 5; SFN-F-EXOs, n = 5) at 48 h (baseline) and 28d after MI to analyze global cardiac function [18, 19] using the Vevo® 2100 high-resolution Imaging System (FUJIFILM VisualSonics Inc., Toronto, Canada) equipped with a 13-24 MHz ultra-high frequency linear array transducer. Images were taken through the left parasternal window while heart rate and rhythm were monitored electrocardiographically. M-mode recordings were obtained by taking short-axis two-dimensional images of the left ventricle at the level of the papillary muscle. According to American Society of Echocardiography guidelines, the global left ventricular (LV) contractile function was measured from the 2D long-axis view and expressed as the ejection fraction (EF, %) during monitoring of heart rate and rhythm. LV fractional shortening (FS, %) refers to the percentage change in end-diastolic (LVEDD) and end-systolic (LVESD) left ventricular diameter as a parameter of how well the left ventricle is contracting itself and therefore reduces the size during systole; it was expressed as (LVEDD-LVESD /LVEDD) × 100. The changes in left ventricular ejection fraction (LVEF, %) were calculated as (LVEF(Day 28) − LVEF(baseline) / LVEF(baseline)) × 100.

### Histological analyses

Myocardial tissue was collected and fixed for 24–48 h in 10% formalin. The samples were dehydrated in an ethanol gradient series, cleared in xylene and embedded in paraffin wax (56 °C). Serial sections with thicknesses ranging from 5 to 7 μm were produced. Sections were deparaffinized in xylene and rehydrated using an ethanol series, as needed. Following PBS washing, slides were mounted with Vectasheild (DBA Italia srl, Segrate, Italy) and images were captured using a fluorescence microscope (DM2500; Leica Microsystems srl, Italy). Capillary density was measured in terms of capillary number/mm^2^. Antigen retrieval for cardiomyocyte size assessment was accomplished using Lab Vision Trypsin enzymatic pretreatment (Thermo Fisher Scientific, Waltham, MA, USA). Before incubating with primary or secondary antibody, slides were rinsed in deionized water and PBS-tween 20 (PBST). Masson’s Trichrome staining with aniline blue (Bio-Optica Milan, Italy) was used for infarct size analysis according to manufacturer's instructions; the infarct scar zone (blue stained) was quantified by ImageJ software and expressed as percentage of left ventricle size, as previously described [32].

Capillary density in the infarct border zone was determined by staining with Wheat Germ Agglutinin (WGA, Invitrogen™, Thermo Fisher Scientific, Waltham, MA, USA) [46] for 30 min at a concentration of 100 μg/mL, as directed by the manufacturer.

Cardiomyocyte cell area was measured by immunofluorescence, labeling cardiomyocytes with a mouse anti-α-Sarcomeric Actinin (α-SA) antibody (Sigma-Aldrich, Merk Life Science S.r.l., Milan, Italy, A7811, lot number: 111M4845 and 055M4827V 1:100) overnight at 4 °C, followed by anti-mouse Alexa Fluor-568 (Abcam, Cambridge, UK) for 90 min at RT. After removing the excess secondary antibody the slides were mounted with DAPI containing Vectasheild (DBA Italia srl, Segrate, Italy). The LV infarct border zone was analyzed in each animal. Cross-sectional cell area was manually measured by ImageJ software and expressed as pixels (Additional file 1).

### Statistical analysis

Graphical depiction and statistical analysis were performed using GraphPad Prism, commercially available software. The data are presented as the mean ± SEM of at least three independent replicates. To compare more than two treatment groups, one -way ANOVA with Tukey post-hoc test was used. To compare two independent groups, Student’s t-test for unpaired values was used. The threshold for statistical significance was chosen at P-value < 0.05.

## Results

### Long-term low-dose SFN affects fibroblast viability in a dose- and time-dependent manner

**Fig. 1:**
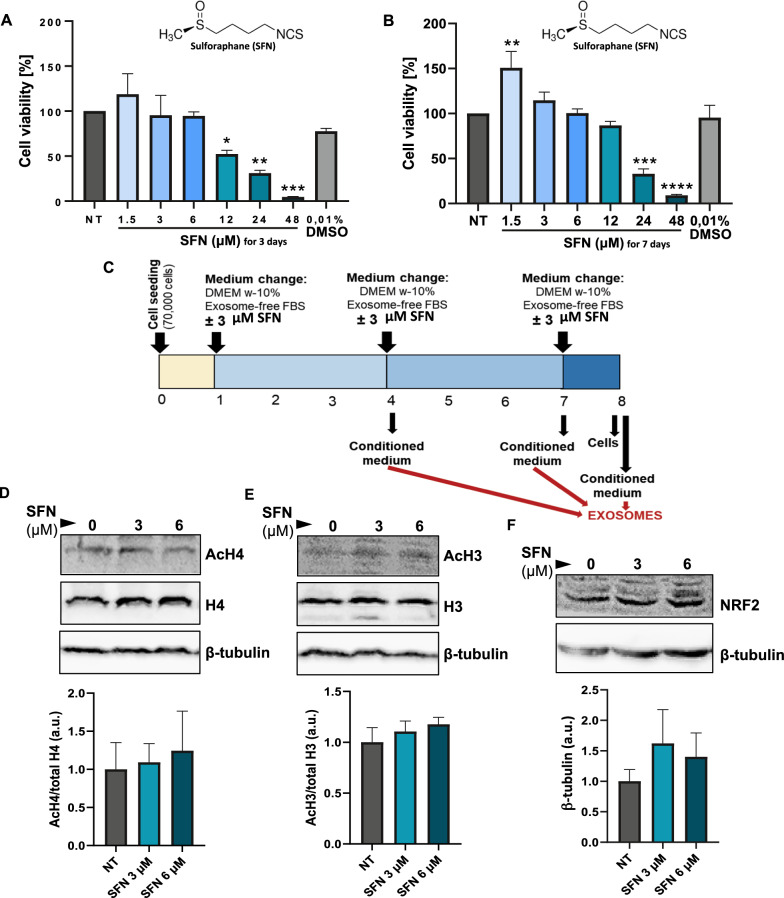
Long-term, dose- and time-dependent effects of sulforaphane on murine NIH/3t3 fibroblasts. **A**-**B** 1,000 NIH/3t3 cells were treated with increasing concentration of sulforaphane (SFN; 1.5 – 48 μM) for 3 (**A**) or 7 days (**B**). Treatment with 0.1% (v/v) DMSO was also performed as a vehicle control. The relative amount of viable cells was measured by MTT assay. Absorbance data are normalized to the control group (Not treated, NT; 0 μM SFN) and represent the mean ± SEM of three independent experiments, each one performed in triplicate replicates. **C** Optimized procedure for SFN treatment of NIH/3t3 cells for exosome extraction: 70,000 NIH/3t3 are cultured on 100 mm cell-culture dishes and treated with ± 3 μM SFN in complete exosome-free culture medium. The treatment is repeated every 3 days and cells are maintained for a total of 7 days; conditioned medium is collected at each medium change (day 3 and 6) and at the end of treatment (day 7). **D**–**F** 70,000 NIH/3t3 cells were treated with ± 3 μM SFN for 7 days in 100 mm cell-culture dishes. Total cell lysate was obtained and the expression of acetylated (Ac) H4 / total H4 histone (**D**), Ac H3/total H3 histone (**E**) and Nrf2/β-tubulin (**F**) was measured by Western blotting. A representative blot is shown for each protein. Densitometric data are normalized to the loading control (β-Tubulin), expressed as relative to the control group (not treated, NT) and represent the mean ± SEM of three independent experiments, each one performed in triplicate replicates. *p < 0.05, **p < 0.01, ***p < 0.001, ****p < 0.0001 vs NT

Depending on the concentration, treatment time, and cell type, SFN has opposing effects on cell death and survival [47]. A preliminary analysis of the dose-time-response relationship of SFN in cultured murine fibroblasts is an important first step in determining the appropriate dose. For 3 and 7 days, cells were treated with SFN concentrations ranging from 1.5 to 48 µM. SFN exhibited no harmful effect on murine fibroblasts at low doses, while increased concentrations lowered cell viability (Fig. 1A-B). SFN treatment of fibroblasts for 3 days reduced cell viability from 12 µM to the highest lethal effect seen at 48 µM. (Fig. 1A). Fibroblasts remained alive after 7 days of treatment with 12 µM SFN (Fig. 1B). Interestingly, 7d treatment with a lower dose of SFN (1.5 µM) boosted cellular metabolic activity, which is consistent with previous findings [47, 48] (Fig. 1B). Conversely, SFN vehicle (DMSO 0.01% (v/v)) had no effect on cell viability at any time point, supporting SFN toxicity at higher doses (Fig. 1A-B). In the light of our findings and earlier research [48], we chose a 3 µM SFN dose and a 7d treatment time with medium changes every 3d to concentrate exosomes (Fig. 1C).

### Long-term low-dose SFN does not alter histone acetylation and Nrf2 levels of fibroblasts

SFN is a dietary dose-dependent inhibitor of class I and II histone deacetylases (HDAC) [24]. As shown in Fig. 1D-E, 7d treatment of NIH/3t3 cells with 3 or 6 µM SFN did not significantly modify histone AcH4 and AcH3 levels. Our findings are consistent with those obtained in human fibroblasts treated with similar dose of SFN [48]. Similarly, 7d treatment of NIH/3t3 cells with 3 or 6 µM SFN did not change Nrf2 expression, as shown in Fig. 1F,

### Long-term low-dose SFN increases exosome release from fibroblasts

**Fig. 2:**
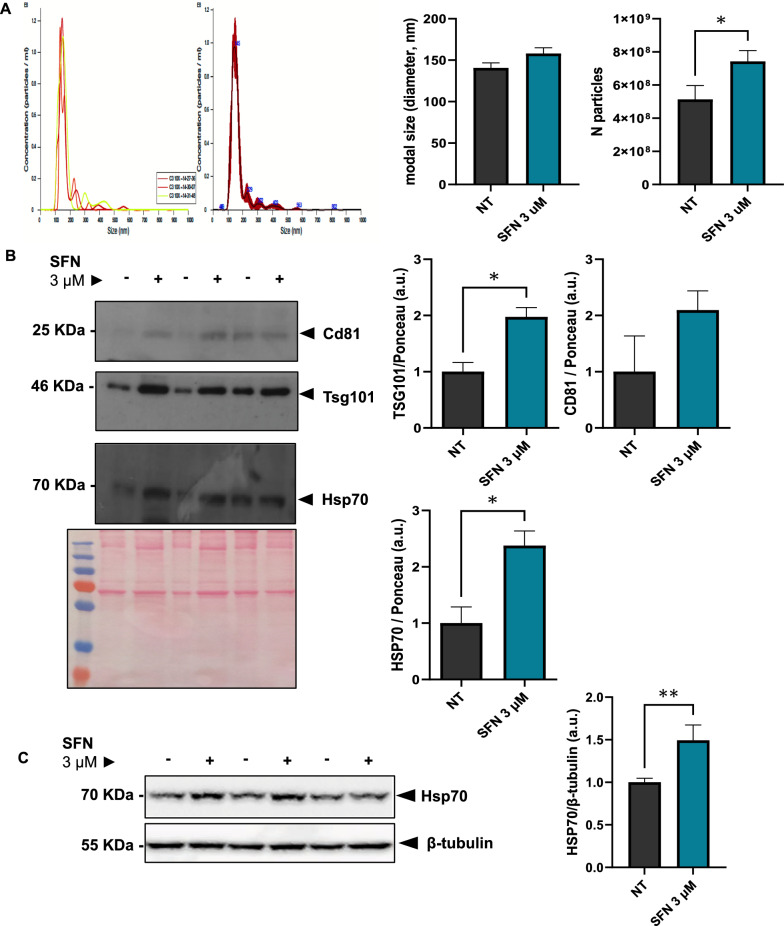
Exosomes secretion from NIH/3t3 fibroblasts is increased by SFN treatment. NIH/3t3 cells were treated with ± 3 μM SFN in exosome-free medium for 7 days in 100 mm cell-culture dishes, medium was collected at day 3, 6 and 7. Exosomes from untreated and SFN-treated (SFN 3 μM) fibroblasts were isolated from the conditioned medium by serial centrifugation and ultracentrifugation. **A** Nanoparticle size distribution and concentration in the controls (NT) and SFN 3 μM pellets were measured by NTA (Nanosight technology). A representative graph of particle distribution is shown. Median particle size and particle number displayed in the bar charts represent the mean ± SEM of three independent experiments, each one analyzed in three acquisitions of one minute each. *p < 0.05. **B** The expression levels of exosome markers (Tsg101, HSP70 and CD81) were measured on the particles’ extracts by Western blotting. A representative blot for each protein and Ponceau S staining are shown. Densitometric data were normalized over Ponceau staining, expressed as relative to the control group (Not treated, 0 μM SFN) and represent the mean ± SEM of three independent experiments. *p < 0.01. **C** The expression of HSP70 was measured on total cell lysates by Western blotting. A representative blot is shown. Densitometric measurements were normalized over for the loading control (β-tubulin), expressed as relative to the control group (Not treated, 0 μM SFN) and represent the mean ± SEM of three independent experiments, each one performed in triplicate replicates. ** p < 0.01

Exosomes were isolated from the conditioned medium of NIH/3t3 fibroblasts after 7 days of treatment with 3 µM SFN using repeated centrifugation and ultracentrifugation. NTA determined the size and concentration of exosomes in accordance with earlier researches [18, 19, 21, 37, 38]. The median exosome size for F-EXOs was 140.5 ± 6.2 nm and 158.0 ± 7 nm for SFN-F-EXOs, as shown in Fig. 2A, which is within the acceptable exosomal size range determined using this technique [49]. Further investigation revealed that the majority of the exosomes released by fibroblasts were between 100 and 160 nm in size, with the number of larger SFN-F-EXOs much higher than F-EXOs of comparable size (Additional file 1: Fig. S1). Indeed, SFN increased fibroblast exosome release by ~ 45% (7.42 ± 0.66 · 108 SFN-F-EXOs versus 5.13 ± 0.84 · 108 F-EXOs per 100 mm plate, p = 0.047). We also determined that SFN treated cells released 1,514 ± 378 exosomes each day, compared to untreated cells, which secreted 1,047 ± 484 exosomes per day.

Isolated and purified exosomes were also identified by the expression of recognized exosomal markers CD81, TSG101 and Hsp70 [12]. Of note, SFN-F-EXOs had larger amounts of TSG101 than F-EXOs for the same concentration of exosomes (~ twofold; p = 0.014). Although CD81 was expressed similarly in SFN-F-EXOs and F-EXOs, levels of Hsp70, an anti-apoptotic [21, 37, 50] and anti-fibrotic [51] exosomal protein, were higher in SFN-F-EXOs (~ 2.4-fold; p = 0.024) (Fig. 2B). Intracellular Hsp70 levels were also significantly elevated in SFN-treated fibroblasts (Fig. 2C).

### SFN-F-EXOs limit AngII-induced growth of murine cardiomyocytes

**Fig. 3:**
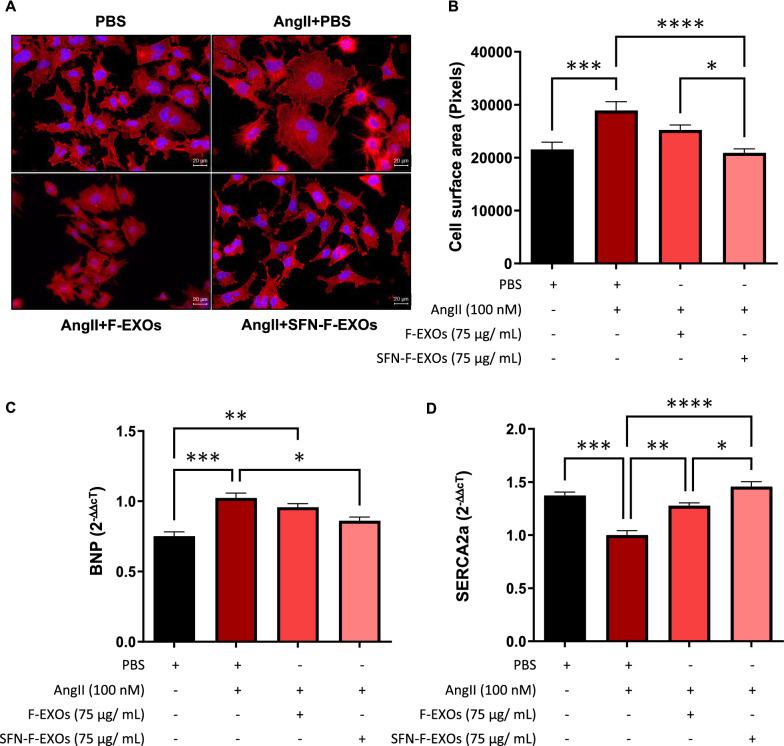
SFN-F-EXOs limit AngII-induced hypertrophy in murine HL-1 cardiomyocytes. HL-1 cardiomyocytes were seeded and treated with or without 100 nM AngII for 12 h. **A** F-EXOs (75 μg/mL) or SFN-F-EXOs (75 μg/mL) were added to each well, and cells were incubated for further 12 h. PBS alone was added as a control. **A**–**B** To measure cell size, HL-1 cell monolayer was fixed, permeabilized and incubated with Phalloidin-Atto550 to fluorescently stain cytoskeletal fibers. Pictures were acquired by fluorescent microscopy (20X magnification); representative pictures are shown (**A**). Cell area was manually measured using ImageJ. Data represent mean cell area ± SEM of three independent experiments, each one analyzed on at least five not-overlapping fields (**B**). *p < 0.05, ***p < 0.001, ****p < 0.0001 **C**, **D** BNP (**C**) and SERCA2a (**D**) levels were quantified by q-RT-PCR; β-actin was employed as housekeeping gene. Expression of the molecular transcript was calculated with the ΔΔCt method and shown as mean 2-ΔΔCt ± SEM of 3 independent experiments. *p < 0.05, **p < 0.01, ***p < 0.001, ****p < 0.0001

AngII, as seen in Fig. 3A-B, increased the cardiomyocytes size compared to resting cells (PBS)(+ 34.3%, p = 0.0002). SFN-F-EXOs counteracted the hypertrophic response induced by AngII (-27.8% vs PBS, p < 0.0001; -17.2% vs F-EXOs, p = 0.02), whereas F-EXOs at identical concentration had no affect on the cell size of stressed cardiomyocytes (Fig. 3A-B).

As shown in Fig. 3C, BNP expression, an established marker of LV hypertrophy induced by AngII [52], was considerably reduced in AngII-stressed HL-1 treated with SFN-F-EXOs (-16%, p = 0.025), whereas a similar amount of F-EXO did not. Then, we assessed the expression of SERCA2a, a sarcoplasmic reticulum Ca2 + ATPase whose up-regulation restricts cardiomyocyte hypertrophy [53], to further analyze the anti-hypertrophic impact. Long-term treatment of AngII-stressed cardiomyocytes with SFN-F-EXOs raised SERCA2a expression compared to PBS (1.46-fold vs PBS, p = 7.18·10^–6^) and to similar amount of F-EXOs (1.14-fold versus F-EXO, p = 0.044), which slightly enhanced SERCA2a levels compared to PBS (p = 0.001) (Fig. 3D).

### SFN-F-EXOs reduce AngII-induced oxidative stress in murine cardiomyocytes

**Fig. 4:**
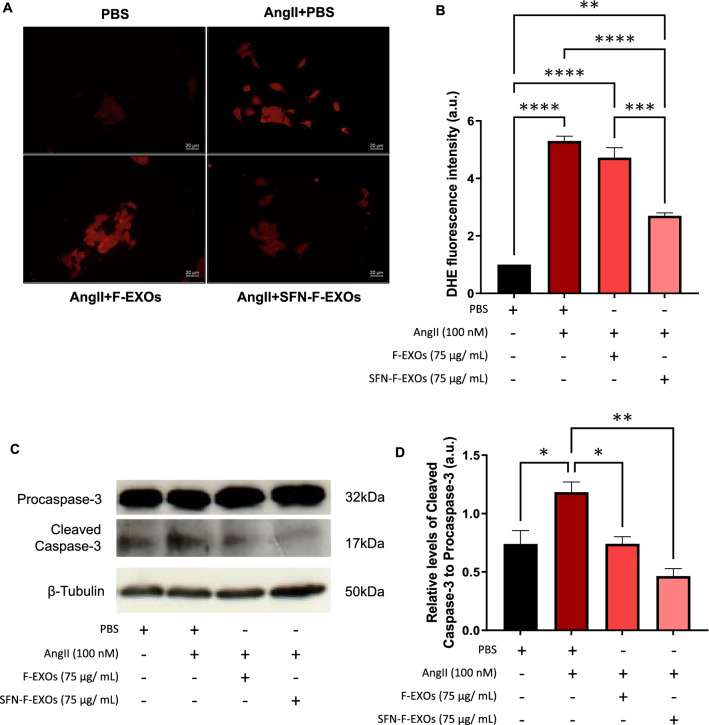
SFN-F-EXOs reduce AngII-induced oxidative stress and apoptosis of murine HL-1 cardiomyocytes. HL-1 cardiomyocytes were seeded in an 8-well chamber slide and treated with or without 100 nM AngII for 12 h. F-EXOs (75 μg/mL) or SFN-F-EXOs (75 μg/mL) were added to each well, and cells were incubated for further 12 h. PBS alone was added as a control. **A**-**B** Superoxide (O2-) generation was detected by DHE staining. Cells were fixed and signal was detected by fluorescent microscopy (20X magnification); a representative picture is shown for each treatment (**A**). Superoxide levels are shown as mean ± SEM of three independent experiments, each one analyzed on at least five not-overlapping fields (**B**). **p < 0.01, ***p < 0.001, ****p < 0.0001. **C**-**D** In order to evaluate apoptosis, procaspase-3 and cleaved caspase-3 levels were measured by Western blotting on HL-1 cells after F-EXO treatment. HL-1 cardiomyocytes were seeded in 6-well plates and treated with or without 100 nM AngII for 12 h. F-EXOs (75 μg/mL) or SFN-F-EXOs (75 μg/mL) were added to each well, and cells were incubated for further 12 h before cell lysis and blotting. PBS alone was employed as a control. A representative blot is shown for each protein (**C**). Protein levels were normalized for the loading control (β-tubulin). Relative levels of cleaved caspase-3 to procaspase-3 are shown in the graph as mean ± SEM of 3 independent experiments (**D**). *p < 0.05, **p < 0.01

AngII causes the production of reactive oxygen species (ROS), which are responsible for the development of heart hypertrophy [54]. Anion superoxide generation was significantly increased in HL-1 cardiomyocytes exposed to AngII (~ fivefold, p < 0.0001), as seen in Fig. 4A-B. The treatment of stressed cardiomyocytes with SFN-F-EXOs reduced the oxidative burst relative to untreated stressed cells (~ twofold decrease vs AngII, p < 0.0001; vs F-EXOs p = 0.001), while ROS levels remained somewhat higher than unstressed cells (vs. PBS p < 0.01). F-EXOs, on the other hand, have no anti-oxidant properties.

### SFN-F-EXOs prevent AngII-induced apoptosis in murine cardiomyocytes

Prolonged exposure to AngII, a mediator of anoxic damage, causes HL-1 cardiomyocytes apoptosis [55]. AngII activated caspase-3 in stressed HL-1, as seen in Fig. 4C-D, which was significantly prevented by both SFN-F-EXOs and F-EXOs.

### Higher uptake of SFN-F-EXOs by murine cardiomyocytes increases histone H4 acetylation

**Fig. 5:**
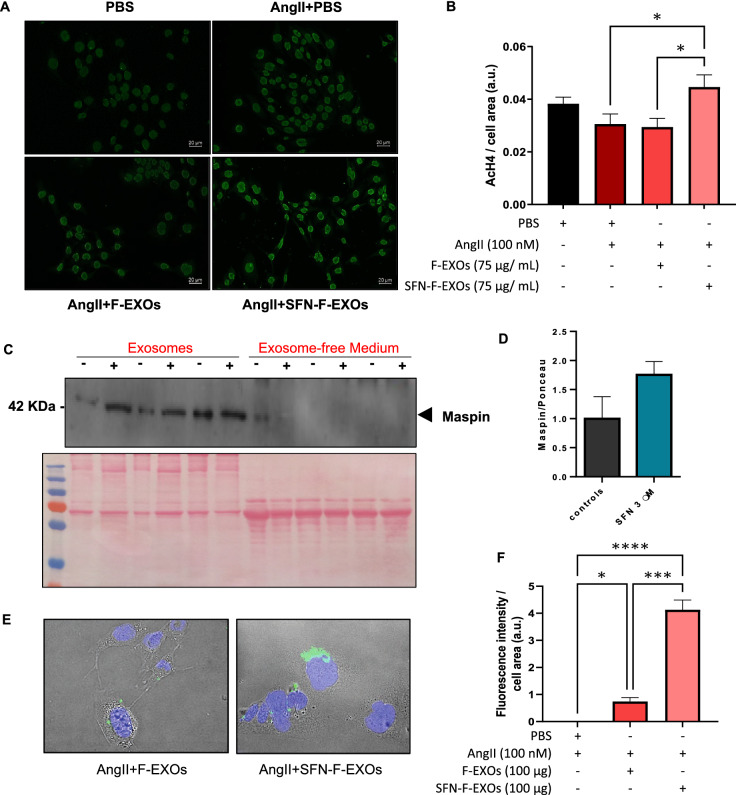
Higher uptake of SFN-F-EXOs by HL-1cardiomyocytes is related to increased histone acetylation. **A**-**B** HL-1 cardiomyocytes were seeded in an 8-well chamber slide and treated with or without 100 nM AngII for 12 h. F-EXOs (75 μg/mL) or SFN-F-EXOs (75 μg/mL) were added to each well, and cells were incubated for further 12 h. PBS alone was added as a control. HL-1 were fixed, permeabilized and immunoprobed using an antibody against AcH4. Signal was detected by fluorescent microscopy (20X magnification); representative pictures are shown (**A**). Fluorescence intensity was normalized for cell area; data are presented as mean ± SEM of three independent experiments, each one analyzed on at least five not-overlapping fields (**B**). *p < 0.05. **C**-**D** Maspin levels in exosomes released from untreated and treated (SFN) NIH/3t3 and in exosome-free culture medium were measured by Western blotting. A representative blot and Ponceau S staining are shown. (**C**). Densitometric data were normalized over Ponceau staining, expressed as relative to the control group (Not treated, 0 μM SFN) and represent the mean ± SEM of three independent experiments (**D**). **E**–**F** In order to evaluate exosomes uptake by cardiomyocytes, HL-1 cardiomyocytes were seeded in an 8-well chamber slide and treated with 100 nM AngII for 12 h, DiA-labelled F-EXOs (100 μg) and SFN-F-EXOs (100 μg) were added to each well. After 3 h, cells were fixed and glass slides mounted in the presence of DAPI (nuclei, blue). Exosome uptake was detected by fluorescent microscopy (20X magnification); representative pictures are shown (**E**). Uptake was quantified as fluorescence intensity of DiA (green, representing the fluorescently-labelled exosomes)/cell surface area. Data are presented as mean ± SEM of three independent experiments, each one analyzed on at least five not-overlapping fields (**F**). *p < 0.05, ***p < 0.001, ****p < 0.0001

Long-term treatment with SFN-F-EXOs, as shown in Fig. 5A-B, raised AcH4 levels in murine cardiomyocytes exposed to AngII (1.46 fold vs PBS, p = 0.048; 1.52-fold vs F-EXOs, p = 0.025), whereas no significant changes were detected in cardiomyocytes treated with F-EXOs. Because histone deacetylase 1 (HDAC1) inhibition lowers pathological cardiac hypertrophy [56] and oxidative stress [57], we assessed the exosomal level of maspin, an HDAC1 inhibitor delivered by exosomes [58, 59]. Maspin was released from fibroblasts into exosomes, as seen in Fig. 5C-D, and its levels were unaffected by SFN treatment. As a result, we postulated that histone acetylation levels may be affected by the extent to which maspin-rich exosomes are incoroporated into cardiomyocytes. Indeed, we found that SFN-F-EXOs were taken up more efficiently by cardiomyocytes grown with (Fig. 5E-F) and without AngII (Additional file 1: Fig. S2) than F-EXOs (5.6-fold vs F-EXOs, p < 0.0002).

### Lower uptake of SFN-F-EXOs by murine cardiac endothelial cells

We studied exosome uptake by MCEC to better understand the cardiomyocyte tropism of exosomes, We found that endothelial incorporation of exosomes was substantially lower than that of cardiomyocytes (34-fold lower in average), but MCEC uptake of SFN-F-EXOs and F-EXOs was equivalent in both experimental settings (Additional file 1: Fig. S3).

### Intramyocardial injection of SFN-F-EXOs attenuates ventricular remodeling in infarcted rats

**Fig. 6:**
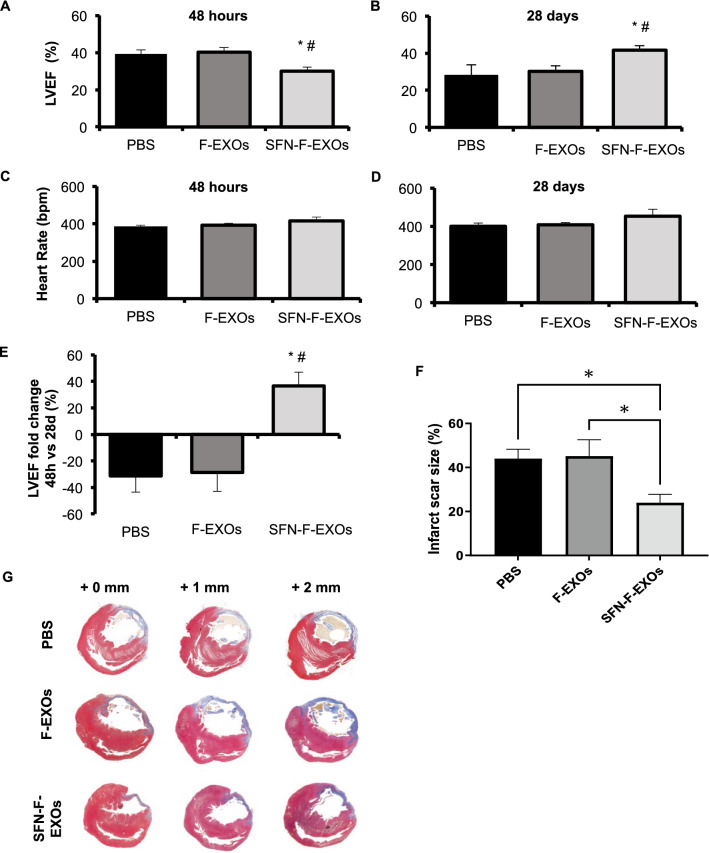
Intramyocardial SFN-F-EXOs limits decay of left ventricular contractility of infarcted heart and reduces infarct scar size in rats. F-EXOs (1*1012 particles in 100 μl PBS) (n = 5) or SFN-F-EXOs (1*1012 particles in 100 μl PBS) (n = 5) or PBS (vehicle, 100 μl) (n = 5) was injected into the myocardium of the left ventricular (LV) region bordering the infarct zone of infarcted rat heart. **A**–**E** Transthoracic echocardiography was performed on anesthetized rats 48 h and 28 days after MI. A,C: Changes of LV ejection fraction (EF, %) and heart rate at 48 h after myocardial infarction (MI). B,D: Changes of LV ejection fraction (EF, %) and heart rate at 28 days after MI. E: Longitudinal fold change (%) of LV Ejection fraction (LVEF) from 48 h to 28 days after MI. Graphs represents the mean ± SEM of five independent animals per group. *p < 0.05 vs PBS, #p < 0.05 vs F.EXOs. **F**–**G** At 28 days after myocardial infarction (MI), heart was explanted, fixed (10% formalin) and paraffin embedded. Serial sections of 7 μm thickness were stained with Masson’s Trichrome staining. Representative images of Masson’s trichrome staining 1 mm apart at three different levels of the left ventricle are shown for each experimental group. Collagen-rich areas (scar tissue) appear in blue and myocardium appears in red (**G**). Percentage of scar size was calculated by normalizing the scar area (blue) for the total area of the left ventricular section. Bar chart represents the mean % infarct scar size ± SEM. Each value results from analysis on three LV sections (**F**). *p < 0.05

The pre-surgery heart rate of all anesthetized rats was 398 ± 18 bpm during ECG monitoring, with no arrhythmia. To test the possible anti-remodeling effect of SFN-F-EXOs or F-EXOs, similar dose of exosomes (1·10^12^ nanoparticles) was injected in the region bordering the infarct zone of rats with permanent LAD ligation. Five rats died suddenly during the first 24 h after surgery: 1/6 (15%) in the SFN-F-EXOs group, 2/7 (30%) in the F-EXOs and PBS groups. At 4 weeks, the surviving infarcted rats showed significant reduction in LVEF after treatment with PBS (-31.33%) or F-EXOs (− 28.7%) (Fig. 6B and E), whereas body weight loss was increased (− 5.5 ± 1%, p < 0.05) as compared to the pre-surgery state. Although SFN-F-EXOs group had significantly lower LVEF than PBS and F-EXOs groups at 48 h after MI (Fig. 6A), we observed better recovery of global cardiac function after intramyocardial injection in infarcted heart of SFN-F-EXOs rats (+ 36.54%) at 28d versus 48 h (baseline) compared to PBS and F-EXOs groups (Fig. 6E), without changing of heart rate (Fig. 6C-D).

**Fig. 7:**
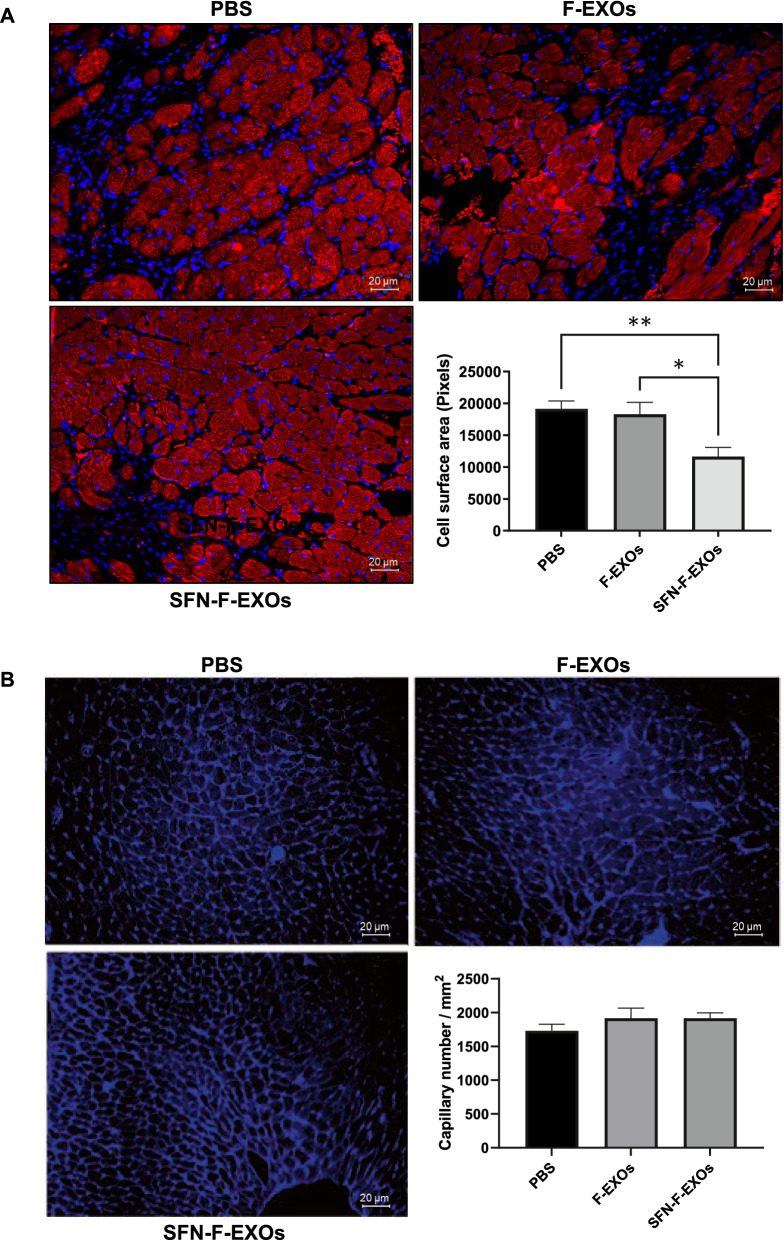
Intramyocardial SFN-F-EXOs reduces cardiomyocytes hypertrophy while it does not affect capillary density in the LV border zone of infarcted rat heart. F-EXOs (1*10^12^ particles in 100 μL PBS) (n = 5) or SFN-F-EXOs (1*10^12^ particles in 100 μL PBS) (n = 5) or PBS (vehicle, 100 μL) (n = 7) was injected into the myocardium of the left ventricular (LV) region bordering the infarct zone of infarcted rat heart. At 28 days after myocardial infarction (MI), heart was explanted, fixed (10% formalin) and paraffin embedded. **A** Serial sections of 5 μm thickness were stained with a mouse anti- α-sarcomeric actinin (SA) antibody and DAPI. Representative images of stained LV infarct border zone are shown for each experimental group. For each heart, five sections of LV infarct border zone for each heart were analyzed. Cross-sectional cell area was measured manually by ImageJ. Values shown in the graphs represent the mean cell surface area ± SEM. *p < 0.05, **p < 0.01. **B** Serial sections of 5 μm thickness were stained with Wheat Germ Agglutinin to measure capillary density. Fluorescent signal was detected by fluorescent microscopy (10X magnification); representative images are shown. Capillary density is expressed as capillary number (circular blue structures)/mm2 and represented in the graph as mean ± SEM

The infarct scar size in rats treated with SFN-F-EXOs was considerably smaller compared to PBS (-46%, p = 0.039) and F-EXOs groups (− 47%, p = 0.044) (Fig. 6F-G), while the infarct scar size in F-EXOs animals was identical to PBS group (Fig. 6F-G). Indeed, a single intramyocardial injection of SFN-F-EXOs resulted in a significant decrease in cardiomyocyte cell area in the infarct border zone (-39.1% vs PBS, p = 0.0078; -36.4% versus F-EXOs, p = 0.027) (Fig. 7A). In contrast, F-EXO intramyocardial injection did not limit post-MI cardiomyocyte hypertrophy. Finally, the number of myocardial capillaries in the infarct border zone was comparable in all groups (Fig. 7B).

## Discussion

Exosome biocompatibility, uptake and function are affected by parent cell type, surrounding microenvironment and exosome-recipient cell interactions [12].

In our study, prolonged chemical conditioning with low-dose SFN, a molecule extracted easily from broccoli and other cruciferous vegetables, increased the release of anti-remodeling exosomes from fibroblast cell lines. Our findings are therapeutically significant since endogenous F-EXOs are ineffective at cardioprotection [15–17], despite the fact that fibroblast-cardiomyocyte communication is critical in post-ischemic myocardium remodeling [4]. Furthermore, our results reveal a novel approach to overcoming present obstacles to the clinical use of allogeneic exosomes for cardioprotection. Indeed, exosome production is dependent on the availability of large numbers of cells, which should release exosomes without compromising their viability or phenotype. Unfortunately, the utilization of autologous cardiac fibroblasts in the hospital setting is severely limited due to the invasive procedure to harvest the myocardium and their low ability to proliferate several times in culture.

We chose the NIH/3T3 cell line for our study because it is similar to adult cardiac fibroblasts [33, 60] and has a high mitotic rate in vitro. In addition, noncardiac fibroblasts are widely used in the production of clinical-grade exosomes for the treatment of many human diseases [34].

Exosomes subpopulations are characterized by a specific size range [61], and our study demonstrates that the majority of fibroblast-derived exosomes are large in size, with low-dose SFN greatly enhancing their release. Importantly, the size of exosomes can influence cargo composition and the route of uptake into recipient cells [62]. TSG101, a component of the endosomal sorting complex necessary for transport machinery that is highly conserved in mice and humans, was shown to be substantially expressed in SFN-F-EXOs and protects ischemic cardiomyocytes from oxidative stress [63]. Furthermore, TSG101-rich exosomes have neuroprotective properties [64]. Hsp70, a molecular chaperone physiologically secreted by exosomes that protects hypoxic cardiomyocytes from apoptosis [21, 37], enriches SFN-F-EXOs. The increase in Hsp70 protein levels in fibroblasts after exposure to SFN, which is known to stimulate heat shock response [65], supports our last finding. However, the regulation of protein cargo sorting and exosome secretion is still a poorly known mechanism. So far, we have ruled out the possibility that alterations in exosome release after chronic SFN 3 µM treatment are due to increasing levels of histone acetylation and Nrf2 expression in viable murine fibroblasts, as opposed to treatment with higher concentrations of SFN [48]. However, TSG101 overexpression may be required for SFN-induced increased exosome secretion [64]. More research into the processes behind SFN-mediated impacts on the biogenesis of fibroblast-derived exosomes is needed.

Since characterizing exosome profile is a prerequisite for understanding their functional impact on recipient cells, we examined their cardioprotective effects. Exosomes released by cardiomyocytes [66], coronary endothelial cells [65], cardiac progenitor cells [18, 19] and stem cells [12] have previously been shown to be naturally cardioprotective in a dose-dependent manner. A similar amount of cardiac fibroblast-derived exosomes, on the other hand, fails to halt cardiac remodeling [18], and may contribute to cardiomyocyte hypertrophy [15]. Similarly, exosomes released from untreated noncardiac fibroblasts do not protect HL-1 cells from AngII-induced damage. A similar amount of SFN-F-EXOs, on the other hand, counteracts AngII-induced growth of murine cardiomyocytes. The anti-hypertrophic effect of SFN-F-EXOs is supported by a considerable reduction in BNP gene expression in AngII-stressed cardiomyocytes, where levels rise in proportion to the degree of myocardial hypertrophy [67]. In terms of the mechanisms underlying the regulation of cardiomyocyte hypertrophic response, it has been established that progressive increase in BNP gene expression is associated with lower SERCA2a levels in post-MI hypertrophy [67]; whereas SERCA2a upregulation has been shown to reduce cardiomyocyte size in vivo [68]. Our findings reveal that SERCA2a expression in stressed cardiomyocytes increases at normal level after treatment with SFN-F-EXOs rather than an equivalent dose of F-EXOs. It is worth noting that a minor rise in SERCA2a expression in stressed HL-1 treated with F-EXOs, while not reaching normal values, it is insufficient to normalize cell size. The current results assume that the anti-remodeling action of exosomes on SERCA2a expression has a threshold dosage. Indeed, only larger levels of SERCA2a were found to provide effective cardioprotection in large animal model of heart failure [69].

The anti-remodeling effect of SFN-F-EXOs piqued our interest and we first investigated if it is related to the reduction of oxidative stress, like with other traditional anti-remodeling therapies [70]. In this regard, we found that superoxide radical anion levels are significantly lower in cardiomyocytes treated with SFN-F-EXOs than in cardiomyocytes treated with a same dose of F-EXOs during persistent AngII exposure. Previous study has found a link between higher SERCA2a expression and lower superoxide anion levels in cardiomyocytes that are more resistant to hypertrophy [69]. Give the importance of AngII in the stimulation of pro-apoptotic signals during cardiomyocyte hypertrophy growth [71], it is not surprising that SFN-F-EXOs improve tolerance to apoptotic oxidative stress. Surprising, a comparable dose of F-EXOs normalizes caspase-3 activation in AngII-stressed cardiomyocytes while leaving oxidative stress and cell size unchanged. Our findings show that inhibiting caspase-dependent signaling is not required to prevent cardiomyocyte enlargement, as previously described [72]. It is more likely that other mechanisms are involved in the anti-hypertrophic response elicited by SFN-F-EXOs.

Notably, the anti-hypertrophic and anti-oxidant actions of SFN-F-EXOs are linked to enhanced histone acetylation in stressed cardiomyocytes. Our results confirm prior research indicating that phytochemicals raise anti-hypertrophic SERCA2a levels by increasing histone acetylation near its promoter region [73]. Moreover, we have previously reported significant cardioprotection associated with enhanced histone acetylation following inhibition of class I histone deacetylase (HDAC) [28], which is known to promote myocardial expression of SERCA2a [74] and to reduce oxidative stress [57]. We cannot rule out the possibility that SFN-F-EXOs may deliver proteins that induce histone acetylation in cardiomyocytes. Maspin, a serpin-like protein that acts as an endogenous intracellular HDAC1 inhibitor [58], is delivered by fibroblast-derived exosomes, to best of our knowledge. Maspin levels are similar in all of our experiments, and its soluble form is undetectable in NIH/3T3 cell culture media, indicating that it is preferentially secreted into exosomes.

Since HDAC inhibitors fully antagonize the hypertrophic program of cardiomyocytes in a dose-dependent manner [75], we assumed that more efficient internalization of SFN-F-EXOs by cardiomyocytes leads to higher levels of histone acetylation; thus, we investigated whether SFN-F-EXOs display better tropism towards cardiomyocytes. Indeed, cardiomyocytes readily taken up SFN-F-EXOs. Interestingly, when cardiac endothelial cells were exposed to a same amount of SFN-F-EXO, they internalized more than 30-fold less than cardiomyocytes under every experimental condition. We cannot rule out the potential that larger exosomal HSP70 levels facilitate higher uptake of large SFN-F-EXOs by cardiomyocytes, has shown that elevated serum Hsp70 levels lead to specific accumulation of Hsp70 on the cardiomyocyte membrane [76]. In fact, Hsp70 has the ability to facilitate endocytosis [77]. Our in vitro data are the first to suggest the use of low-dose SFN as chemical agent eliciting the release of substantial amount of cardioprotective exosomes with high cardiomyocyte tropism from noncardiac fibroblast cell line.

Purified SFN-F-EXOs, when injected into the infarct border zone of the rat heart, improve the progressive worsening of global cardiac function within four weeks following MI without altering heart rate or increasing body weight loss, a well-known reliable early noninvasive marker of HF [78]. Intramyocardial injection of SFN-F-EXOs reduces scar size while limiting cardiomyocyte size in the infarct border zone 28 days after LAD ligation. In contrast, a similar amount of purified F-EXOs injected intramyocardially has no anti-remodeling effect or improves cardiac contractility. Finally, both experimental groups have equal capillary density in the LV border zone. Although the lack of angiogenic effect in exosome-treated hearts may be explained by the weak endothelial tropism of fibroblast-derived EXOs, myocardial hypertrophy without changes in capillary density is expected. Indeed, despite increasing cardiomyocyte cell size, the number of capillaries per myocyte may remain unaltered, rendering the myocardium vulnerable to insufficient oxygenation [79] and larger scar formation.

### Limitations of the study and perspectives

Our initial proof of concept study will support more in-depth research into the molecular mechanisms behind the effect of low-dose SFN on exosome biogenesis. The first characterization of the SFN-F-EXOs phenotype provided by our study strongly encourages future thorough investigations of exosomal cargo to establish quantitative production standards for clinical use. Additional experiments should be done to assess the clinical value of prolonged systemic administration of SFN-F-EXOs in large animal models of MI during hemodynamic monitoring, in order to develop a new therapeutic strategy for HF prevention that is easily transferable to the hospital setting, and even to validate a risk score model for predicting the cardiovascular outcome after treatment.

### Conclusions

We demonstrated that continuous low-dose SFN treatment is a novel chemical-based method for producing anti-remodeling exosomes from a noncardiac fibroblast cell line. SFN increases the tropism of noncardiac exosomes towards cardiomyocytes, which higher internalization increases AcH4 levels, induces SERCA2a expression, lowers apoptotic oxidative stress and hinders the hypertrophic response. Our approach could pave the way for safe and cost-effective large-scale production of natural cardioprotective exosomes for therapeutic use without the need for vector-mediated gene editing of allogeneic fibroblasts to limit immunogenicity or for biomimetic exosomes to improve the distribution to the target cell. Furthermore, the cardioprotective procedure requiring a single myocardial injection of ready to use stable allogeneic SFN-F-EXOs, previously stored at − 80 °C avoiding freezing–thawing [80], can serve as good emergency cell-free treatment to rescue a damaged heart without waiting for the time-consuming autologous exosome generation, especially in older and frail patients.

## Supplementary Information

**Additional file 1:** **Figure S1. Particle analysis of F-EXOs from NIH/3t3 fibroblasts upon SFN treatments how a clear prevalence of large exosome sizes.** NIH/3t3 cells were treated with ±3 μM medium was collected at day 3, 6 and 7. Exosomes from untreated and SFN-treated (SFN 3μM) fibroblasts were isolated from the conditioned medium by serial centrifugation and ultracentrifugation. Nanoparticle size distribution measured by NTA (Nanosight technology). Particles subpopulations were determined in terms of particles size (45-75 nm; 75-100 nm; 100-160 nm); particles concentrations in each subpopulation are presented as mean±acquisitions of one minute each. *p<0.05 vs NT. **Figure S2.** Higher uptake order to evaluate exosomes uptake by cardiomyocytes, HL-1 cardiomyocytes were seeded in an 8-well chamber for 24 h before adding DiA-labelled F-EXOs (100 μg) and SFN-FEXOs (100 μg) to each well. After 3 h, cells were Exosome uptake was detected by fluorescent microscopy (20X magnification); Uptake was quantified as fluorescence intensity of DiA (green, representing the fluorescently-labelled exosomes)/cell surface area. Data are presented on at least five not-overlapping fields (F). **p<0.01, ***p<0.001, ****p<0.0001. **Figure S3. SFN-F-EXOs are poorly taken up by murine cardiac endothelial cells.** Murine cardiac endothelial cells (MCEC) were seeded in an 8-well chamber slide and treated with 100 nM AngII for well, and cells were incubated for further 3 h. Cells were fixed with PFA and glass slides mounted in the presence of DAPI (nuclei, blue). Exosome uptake was detected by fluorescent microscopy (20X magnification). Uptake fluorescently-labelled exosomes)/cell surface area.Data are presented as mean±SEM of three independent experiments, each one analyzed on at least five not-overlapping fields. HL-1 uptake is presented on the right for the comparison of the uptake magnitudes.

## Data Availability

The datasets during and/or analysed during the current study available from the corresponding author on reasonable request.

## Abbreviations

α-SA: α-Sarcomeric Actinin
AcH3: Acetylated histone H3
AcH4: Acetylated histone H4
AngII: Angiotensin II
BNP: Brain natriuretic peptide
DHE: Dihydroethidium
DMEM: Dulbecco’s Modified Eagle’s Medium
DMSO: Dimethyl sulfoxide
EXOs: Exosomes
F: Fibroblast
FBS: Fetal bovine serum
F-EXOs: Fibroblast-derived exosomes
HDAC1: Histone deacetylase 1
HF: Heart failure
Hsp70: Heat shock protein 70
LAD: Left anterior descending coronary artery
LV: Left ventricular
LVEDD: Left ventricular end-diastolic diameter
LVEF: Left ventricular ejection fraction
LVESD: Left ventricular end-systolic diameter
LVFS: Left ventricular fractional shortening
MCEC: Murine cardiac endothelial cells
MI: Myocardial infarction
Nrf2: Nuclear factor erythroid 2–related factor 2
PBS: Phosphate buffered saline
PBST: PBS-tween 20
PFA: Paraformaldehyde
ROS: Reactive oxygen species
SERCA2a: Sarcoplasmic/endoplasmic reticulum Ca^2+^ ATPase 2a
SFN: Sulforaphane
SFN-F-EXOs: SFN-induced F-EXOs
TSG101: Tumor susceptibility gene 101
