# Supplementary material for: Cardiomyocyte-targeting exosomes from sulforaphane-treated fibroblasts affords cardioprotection in infarcted rats
Source: J Transl Med. 2023 May 9;21:313. doi: 10.1186/s12967-023-04155-x (PMC10169450; doi:10.1186/s12967-023-04155-x)
Supplement: Supplementary file 1 — Additional file 1: Figure S1. Particle analysis of F-EXOs from NIH/3t3 fibroblasts upon SFN treatments how a clear prevalence of large exosome sizes. NIH/3t3 cells were treated with ±3 μM medium was collected at day 3, 6 and 7. Exosomes from untreated and SFN-treated (SFN 3μM) fibroblasts were isolated from the conditioned medium by serial centrifugation and ultracentrifugation. Nanoparticle size distribution measured by NTA (Nanosight technology). Particles subpopulations were determined in terms of particles size (45-75 nm; 75-100 nm; 100-160 nm); particles concentrations in each subpopulation are presented as mean±acquisitions of one minute each. *p<0.05 vs NT. Figure S2. Higher uptake order to evaluate exosomes uptake by cardiomyocytes, HL-1 cardiomyocytes were seeded in an 8-well chamber for 24 h before adding DiA-labelled F-EXOs (100 μg) and SFN-FEXOs (100 μg) to each well. After 3 h, cells were Exosome uptake was detected by fluorescent microscopy (20X magnification); Uptake was quantified as fluorescence intensity of DiA (green, representing the fluorescently-labelled exosomes)/cell surface area. Data are presented on at least five not-overlapping fields (F). **p<0.01, ***p<0.001, ****p<0.0001. Figure S3. SFN-F-EXOs are poorly taken up by murine cardiac endothelial cells. Murine cardiac endothelial cells (MCEC) were seeded in an 8-well chamber slide and treated with 100 nM AngII for well, and cells were incubated for further 3 h. Cells were fixed with PFA and glass slides mounted in the presence of DAPI (nuclei, blue). Exosome uptake was detected by fluorescent microscopy (20X magnification). Uptake fluorescently-labelled exosomes)/cell surface area.Data are presented as mean±SEM of three independent experiments, each one analyzed on at least five not-overlapping fields. HL-1 uptake is presented on the right for the comparison of the uptake magnitudes. [file 12967_2023_4155_MOESM1_ESM.pdf]

Additional file 1: Figure S1

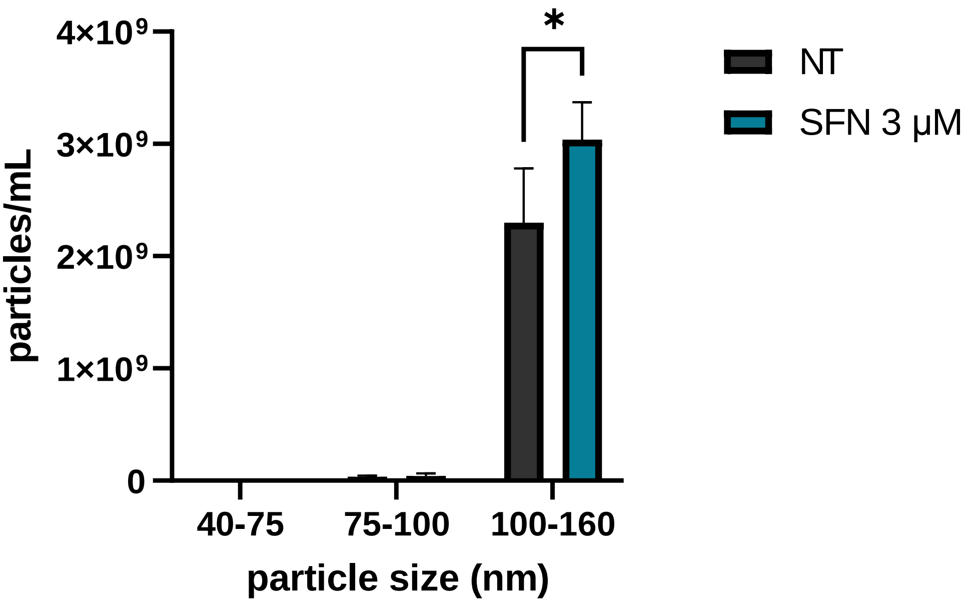

Additional file 1: Figure S1 Particle analysis of F-EXO from NIH/3t3 fibroblasts upon SFN treatment show a clear prevalence of large exosome sizes. NIH/3t3 cells were treated with ± 3 μM SFN in exosome-free medium for 7 days in 100 mm cell-culture dishes, medium was collected at day 3, 6 and 7. Exosomes from untreated and SFN-treated (SFN 3 μM) fibroblasts were isolated from the conditioned medium by serial centrifugation and ultracentrifugation. Nanoparticle size distribution and concentration in the controls (NT) and SFN 3 μM pellets were measured by NTA (Nanosight technology). Particles subpopulations were determined in terms of particles size (45-75 nm; 75-100 nm; 100-160 nm); particles concentrations in each subpopulation are presented as mean ± SEM of three independent experiments, each one analyzed in three acquisitions of one minute each. *$p<0.05$ vs NT.

Additional file 1: Figure S2

Additional file 1: Figure S1

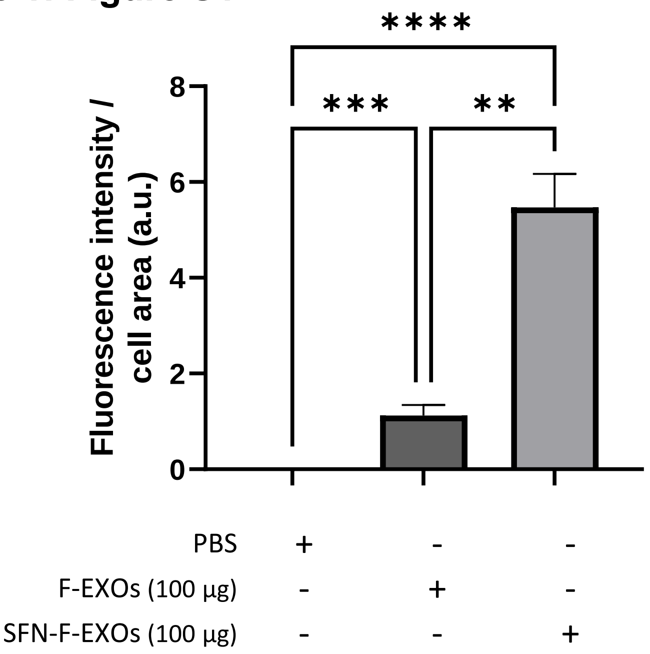

Additional file 1: Figure S2. Higher uptake of SFN-F-EXOs by HL-1 cardiomyocytes in resting conditions. In order to evaluate exosomes uptake by cardiomyocytes, HL-1 cardiomyocytes were seeded in an 8-well chamber for 24h before adding DiA-labelled F-EXOs (100 µg) and SFN-F-EXOs (100 µg) to each well. After 3h, cells were fixed and glass slides mounted in the presence of DAPI (nuclei, blue). Exosome uptake was detected by fluorescent microscopy (20X magnification); Uptake was quantified as fluorescence intensity of DiA (green, representing the fluorescently-labelled exosomes)/cell surface area. Data are presented as mean ± SEM of three independent experiments, each one analyzed on at least five not-overlapping fields (F). **$p<0.01$, ***$p<0.001$, ****$p<0.0001$

Additional file 1: Figure S3

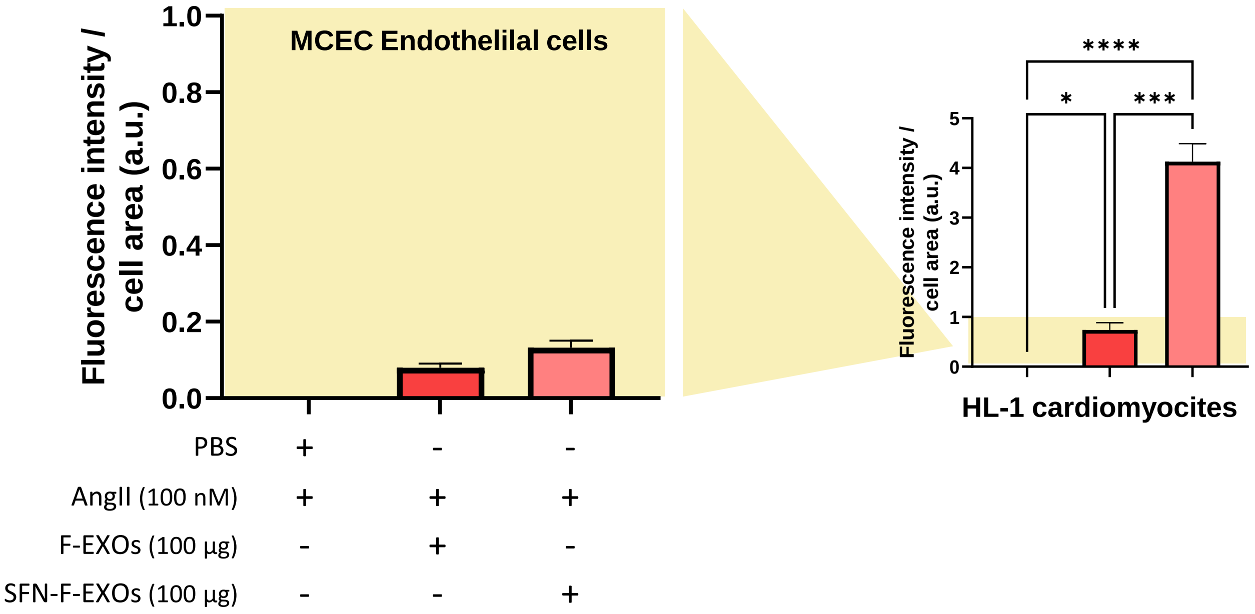

Additional file 1: Figure S3. SFN-F-EXOs are poorly taken up by murine cardiac endothelial cells. Murine cardiac endothelial cells (MCEC) were seeded in an 8-well chamber slide and treated with 100 nM AngII for 12h**.** 100ug DiA-labelled F-EXOs and SFN-F-EXOs were added to each well, and cells were incubated for further 3h. Cells were fixed with PFA and glass slides mounted in the presence of DAPI (nuclei, blue). Exosome uptake was detected by fluorescent microscopy (20X magnification). Uptake was quantified as fluorescence intensity of DiA (green, representing the fluorescently-labelled exosomes)/cell surface area. Data are presented as mean ± SEM of three independent experiments, each one analyzed on at least five not-overlapping fields. HL1 uptake is presented on the right for the comparison of the uptake magnitudes.
